# The Efficacy of Using Video Laryngoscopy on Tracheal Intubation by Novice Physicians

**DOI:** 10.22038/ijorl.2020.43797.2447

**Published:** 2021-01

**Authors:** Maryam Ilbagi, Mohammad Nasr-Esfahani

**Affiliations:** 1 *Department of Emergency Medicine, Al-Zahra Medical Center, School of Medicine* *.* * Isfahan University of Medical Sciences, Isfahan, Iran.*

**Keywords:** Direct laryngoscopy, Hemodynamic, Laryngeal intubation, Video laryngoscopy

## Abstract

**Introduction:**

The first successful attempt at tracheal intubation with minimal complications is crucial for emergency physicians. The aim of this study was to compare endotracheal intubation using video laryngoscopy versus direct laryngoscopy in the emergency department by emergency medicine residents.

**Materials and Methods:**

In this randomized clinical trial, 70 patients requiring laryngeal intubation were randomly enrolled in direct and video laryngoscopy groups. The first attempt success rate, frequency of attempts, complications, and hemodynamic changes after laryngoscopy were assessed. The data were analyzed using the Chi-square, independent t-test, and Fisher's exact test.

**Results:**

The results showed a significant increase in heart rate, as well as systolic and diastolic blood pressure after both direct and video laryngoscopy (P<0.001). However, this increase was more severe in the video laryngoscopy group (P<0.001).

**Conclusion:**

Although the use of both devices had similar success rate, if orotracheal intubation is performed by a novice emergency medicine residents, direct laryngoscopy causes fewer hemodynamic effects on patients, compared to video laryngoscopy.

## Introduction

Orotracheal intubation (OTI) with laryngoscopy is one of the important treatment measures in patients requiring respiratory support (1). The OTI is a safe and secure way to create and maintain an airway. Some OTI indications include high concentration oxygen administration, cardiac arrhythmias, cerebral edema, hypoxia, hypoventilation, decreased level of consciousness, Glasgow Coma Scale score less than 8, lack of reflexes or reduced gag reflex, prevention of secondary respiration problems, head and neck trauma, anesthesia, and surgeries. 

Direct laryngoscopy is the most common method of OTI, which is associated with potential complications (2). Stimulation of the sympathetic system and hemodynamic changes, such as increased blood pressure, increased heart rate, dysrhythmia, stimulation of cough reflex, increased intracranial pressure, increased intraocular pressure, and stimulation changes in the electroencephalogram, are among the OTI complications (3). Other complications of laryngoscopy include airway trauma, laryngospasm, bronchospasm, wrong tracheal intubation in the esophagus, vomiting, and aspiration (4,5).

In a direct laryngoscopy, the pharynx and tube all will place in the same direction with the pressure and tension on the mouth. The laryngoscope blade is designed to hold the tongue back and keep the head in the proper position so that vocal folds can be observed directly (6).

Video laryngoscopy(this technology allows the expert to see the tube on a video screen while it is being inserted) is a substitute for direct laryngoscopy, which provides the physician with an appropriate indirect view without excessive pressure on the mouth and pharynx (7). Previous studies performed a comparison of direct and video laryngoscopy with contradictory results (1,7-11). In some studies, it has been reported that the use of video laryngoscopy is associated with an increased success rate in the first attempt to intubate and reduced misplacement of the tracheal tube in the esophagus (8,12-16).

However, other studies revealed no difference between the two approaches regarding the success rate of the first-attempt intubation (7,17-20). One of the intubation complications is a decrease in the blood oxygen saturation level. Some studies have reported that the percentage of blood oxygen saturation in direct laryngoscopy is higher than that in the video laryngoscopy (9,16), whereas there is no difference between the two methods in terms of the hypoxia occurrences in other studies (4,7).

Stimulation of the sympathetic system in laryngoscopy can lead to hemodynamic changes in patients (10,21,22). The results of previously conducted studies are also contradictory in this area. Some studies have argued that direct laryngoscopy leads to more severe hemodynamic changes in patients (21,23), while other studies have not observed a significant difference between the two methods in this regard (1,11,24).

The present study aimed at investigating the risk of complications and failure of using two methods of direct and video laryngoscopy on patients, who underwent endotracheal intubation by emergency medicine residents. The results were assessed to evaluate the benefits and risks of using these two methods in the target population.

## Materials and Methods

This clinical trial was performed on patients undergoing endotracheal intubation from 2016 to 2018 at Alzahra University Hospital, Isfahan, Iran. After obtaining the approval from the Ethics Committee of the Isfahan Medical School, Isfahan, Iran (IR.MUI.REC. 1396.3.543), seventy patients were recruited for the study according to the sample size calculation and based on inclusion and exclusion criteria.

Inclusion criteria were: 1) suitability for undergoing endotracheal intubation, 2) age range of 18-65 years, 3) lack of anatomical problems in the neck and trachea, 4) no drug addiction, and 5) willingness to participate in the study. 

On the other hand, the patients with cardiac arrest during laryngoscopy and difficult intubation during attempts to intubate, as well as those in situations when intubation was prohibited (i.e., unstable spinal cord injury) were excluded from the study.It is worth mentioning that the patients were not obese. According to the American Society of Anesthesiologists' Classification of Physical Health, the selected patients for intubation were in class II based on their health; however, they were not more than II according to the Mallampati classification. All participants had a thyromental distance equal or more than 6cm. Subsequently, Participants were randomly assigned to one of the two groups, based on sequential numbers. They received fentanyl (3 mcg/kg IV) after pre-oxygenation for 3 minutes. Anesthesia induction was performed with injections of etomidate (0.3 mg/kg IV) and succinylcholine (1.5 mg/kgIV). Tube sizes of 7 and 8 were selected for females and males, respectively; moreover, blood pressure, pulse oximetry, and ECG were monitored.

The first group was subjected to direct laryngoscopy using a Macintosh laryngoscope (No. 3), and the second group underwent video laryngoscopy using a Glide Scope with a blade No. 3. All patients were monitored by pulseoximetry, sphygmomanometer, and capnography before intubation. Afterward, they were attached to the ventilator after intubation. All interventions in both groups were performed by the second year Emergency Medicine Resident who had sufficient skill in using direct and video laryngoscopes.

The length of the laryngoscopy- from the time the laryngoscope entered the mouth until the tube passed the vocal cords- was measured in seconds. The frequency of attempts and the success rate of laryngoscopy at the first attempt to intubate was also recorded in this study. In addition, heart rate, as well as systolic and diastolicblood pressure, were measured and recorded in all patients before, during, and after intubation.

Respiratory complications of intubation (laryngospasm) and direct complications of intubation (reduced blood pressure and blood oxygen saturation, aspiration, and wrong intubation) were also assessed and recorded in this study. Moreover, time measurement and recording of the patient variables were performed by the nurse attending the clinic. After collecting the data, descriptive statistical tests (relative frequency, mean, and standard deviation), and inferential statistical tests including the Chi-square, independent t-test, and Fisher's exact test were analyzed using SPSS software (version 18).

## Results

This study investigated 70 patients in two groups of 35 cases per group. Table 1 tabulates the mean values of age and weight, as well as the frequency of the patients’ gender. The percentage of success in the first attempt to intubate in the direct and video laryngoscopy groups were 62.9% and 71.4%, respectively, which showed no statistically significant difference between the two groups in this regard (P=0.44) (Table.1). In the direct laryngoscopy group, 62.9% of the patients were intubated at the first attempt, 31.4% in the second attempt, and 5.7% in the third attempt. On the other hand, in the video laryngoscopy group 74.3%, 22.9%, and 2.9% of the patients were intubated in the first, second, and third attempts, respectively, which was not significant between the two groups (P=0.56) (Table 1).

**Table 1:** Demographic characteristics of the two groups

	**Video laryngoscopy**	**Direct laryngoscopy**	**P-value**	**Test**
Age	45.74±11.289	45.31±11.172	0.87	t-test
Weight	70.11±10.566	70.89±10.613	0.76	t-test
Gender				
Female	15 (42.9%)	19 (54.3%)	0.33	Chi-square
Male	20 (57.1%)	16 (45.7%)		
Success in the first attempt to intubate				
Yes	25 (71.4%)	22 (62.9%)	0.44	Chi-square
No	10 (28.6%)	13 (37.1%)		
Number of attempts				
1	26 (74.3%)	22 (62.9%)	0.56	Fisher's exact test
2	8 (22.9%)	11 (31.4%)		
3	1 (2.9%)	2 (5.7%)		

A summary of intubation complications is presented in Table 2. In the direct and video laryngoscopy groups, 8.6% and 14.3% of the patients suffered from aspiration and vomiting, respectively, which was not statistically significant (P=0.71) (Table.2). In this study, 11.4% and 2.9% of the patients who underwent direct and video laryngoscopy had misplacement of the tube in the esophagus, respectively, which was not statistically significant (P=0.35) (Table.2). Furthermore, in assessing reduced oxygen saturation of the patients during the intubation, 14.3% and 8.6% of the patients in the direct and video laryngoscopy groups suffered from intubation; however, the difference between the two groups was not significant in this regard (P=0.71) (Table.2).

**Table 2:** Complications in the two groups

	**Direct laryngoscopy**	**Video laryngoscopy**	**P-value**	**Test**
Aspiration and vomiting				
Yes	3(8.6%)	5(14.3%)	0.71	Fisher's exact test
No	32(91.4%)	30(85.7%)		
Misplaced tube in the esophagus				
Yes	4(11.4%)	1(2.9%)	0.35	Fisher exact test
No	31(88.6%)	34(97.1%)		
Reduced blood oxygen				
Yes	5(14.3%)	3(8.6%)	0.71	Fisher exact test
No	30(85.7%)	32(91.4%)		

There was no significant difference between the two groups regarding mean systolic and diastolic blood pressure, as well as heart rate in patients before laryngoscopy (Table.3). The mean systolic blood pressure after laryngoscopy increased from 118.2 to 139.23 mmHg and from 120.51 to 158.89 mmHg in the direct and video laryngoscopy group, respectively. This showed a significant change in the systolic blood pressure in each group (P<0.001) (Table.3). Moreover, the mean diastolic blood pressure increased from 72.03 to 93.69 mmHg and from 70.83 to 116.03 mmHg after direct and video laryngoscopy in patients, respectively, which revealed a statistically significant change in diastolic blood pressure in both groups (P<0.001) (Table.3). Furthermore, the mean heart rate of the patients after laryngoscopy in the direct and video laryngoscopy groups increased from 73.91 to 83.71 and from 72.80 to 92.86 beats per minute, respectively, which indicated a statistically significant change in each group (P<0.001) (Table.3).

**Table 3:** Systolic and diastolic blood pressure as well as heart rate in two groups

**Independent t-test results**	**Direct laryngoscopy**		**Video laryngoscopy**		**Group** **Stage**	**Variable **
	**Standard deviation**	**Mean**	**Standard deviation**	**Mean**		
P=0.49	14.104	118.20	13.783	120.51	Before	Systolic blood pressure
P<0.001	13.469	139.23	17.182	158.89	After	
	P<0.001		P<0.001		Paired t-test result	
P=0.58	8.322	72.03	9.802	70.83	Before	Diastolic blood pressure
P<0.001	12.497	93.69	17.269	116.03	After	
	P<0.001		P<0.001		Paired t-testresult	
P=0.55	8.038	73.91	7.677	72.80	Before	Heart rate
P<0.001	9.173	83.71	6.665	92.86	After	
	P<0.001		P<0.001		Paired t-testresult	

The mean value of systolic blood pressure changes observed in the video laryngoscopy group was significantly higher than that in the direct laryngoscopy, which was statistically significant (38.37 versus 21.03 mm Hg) (P<0.001) (Table.4). It was also observed that the video laryngoscopy exerted more severe changes in diastolic blood pressure, compared to the direct laryngoscopy, which was statistically significant (45.02 vs. 21.66 mmHg) (P<0.001) (Table.4).

The mean value of heart rate changes was also more severe in video laryngoscopy, compared to the direct laryngoscopy (20.06 versus 9.80) (P<0.001) (Table.4).

**Table 4:** Changes in systolic and diastolic blood pressure as well as heart rate in two groups

**Independent t-test results**	**Direct laryngoscopy**		**Video laryngoscopy**		**Variable **
	**Standard Deviation**	**Mean**	**Standard Deviation**	**Mean**	
P<0.001	6.780	21.03	14.053	38.37	Systolic Blood Pressure
P<0.001	8.102	21.66	14.911	45.20	Diastolic Blood Pressure
P<0.001	3.620	9.80	4.946	20.06	Heart rate
					

## Discussion

This clinical trial aimed at comparing hemodynamic response and complications of direct and video laryngoscopy in patients requiring intubation. The main finding of this study was the presence of more severe hemodynamic responses in the video laryngoscopy group, compared to the direct laryngoscopy group. The mean values of systolic and diastolic blood pressure, as well as heart rate after intubation, increased more significantly in the video laryngoscopy group, compared to the direct laryngoscopy group, which was the opposite of the expected result. 

Similar results were obtained in a study conducted by Naghibi et al. (25) in 2014. The aforementioned study assigned 60 candidates over 65 years old into two groups of direct and video laryngoscopy. The mean difference before and after systolic and diastolic blood pressure was significantly higher in the video laryngoscopy group, compared to the other group. Furthermore, they concluded that the hemodynamic response was more severe in the video laryngoscopy group, compared to the direct laryngoscopy. 

Arulkumaran et al. (13) conducted a review in 2018 to compare the effects of video and direct laryngoscopy complications. They showed that the incidence of arterial blood pressure drop during intubation was greater in video laryngoscopy, compared to the direct laryngoscopy, which was in contrast to the results of other previous studies.It is stated that the above-mentioned effect is probably due to sedative and vasopressor agents used during intubation; however, the interpretation of this result is not possible in isolation.

Contrary to the mentioned studies, other studies reported that direct laryngoscopy led to more hemodynamic responses in patients, compared to video laryngoscopy (23,26). In the same vein, Barman et al. in 2017 evaluated direct and video laryngoscopy in patients undergoing coronary artery bypass graft surgery (23). According to the results, there was no difference between the two groups in terms of the mean heart rate before and after intubation; however, the mean systolic and diastolic blood pressure after intubation increased in both groups so that the increase was significantly higher in the direct laryngoscopy group, compared to the other group. In addition, Wie et al. in 2016 (26) reported that intubation with video laryngoscopy compared to direct laryngoscopy led to a reduction in hemodynamic responses in patients. In addition to the above results, several studies have not observed any difference in hemodynamic responses among patients undergoing direct and video laryngoscopy (11,27). Abdelgawad et al. in 2015 (11) investigated 60 candidates for intubation who were assigned into three groups of direct laryngoscopy (n=1) and different types of video laryngoscopy (n=2). Subsequently, the candidates in each group were divided into two subgroups with normal and hypertensive blood pressure. 

Considering the patients with normal blood pressure, there was no difference among the three groups regarding hemodynamic response; however; in hypertensive patients, the hemodynamic response was significantly higher in direct laryngoscopy group, compared to the other two groups, which indicated a more severe hemodynamic response in hypertensive patients. Similarly, Pournajafian et al. in 2014 (27) assessed 95 patients and reported no difference between direct and video laryngoscopy regarding the severity of the hemodynamic response. 

The contradiction in the results of the previously conducted studies can be explained by the sympathetic system stimulation during the two steps of laryngoscopy and intubation. The first step during laryngoscopy is to create a proper look at the glottis and vocal cords, and the second step is when the tube passes through the vocal cords. This depends on the pressure exerted on surrounding tissues, time needed for intubation, and repeated frequencies which all are affected by the surgeon’s skill (6).

Another finding of the present study was the lack of difference between both groups in the success rate of the first attempt to intubate and the frequency of attempts. 

The results of this study were in line with the findings reported in other studies. Ducharme et al.(28) in 2017 showed that 66.7% and 62.5% of the patients in the direct and video laryngoscopy groups were successfully intubated in the first attempt, and the difference between the two groups was not statistically significant. In addition, a systematic review performed by Huang et al.(29) in 2017 compared direct and video laryngoscopy in patients at the ICU. It was reported that the success rate of intubation in the first attempt in the video laryngoscopy method was equal to the direct laryngoscopy, and there was no evidence for the superiority of the video laryngoscopy. In two meta-analyses carried out by Bhattacharjee (19) (2018) and Lewis (7) (2017), the use of video laryngoscopy was associated with a decrease in the rate of misplaced intubation in the esophagus; however, the success rate in the first attempt of intubation and the overall intubation success rate had no increase with direct laryngoscopy.

Nevertheless, Arulkumaran et al. (13) conducted a meta-analysis in 2017 and stated that the use of video laryngoscopy by less experienced physicians increased the success rate of intubation in the first attempt. This reflects the dependence of intubation results on personnel experience. Another finding of the present study is the lack of difference between the two laryngoscopy methods regarding complications, such as hypoxia, misplaced intubation in the esophagus, aspiration, and vomiting. 

Arulkumaran(13) also revealed no significant difference between the two methods in terms of hypoxia and aspiration of stomach contents; however, in the video laryngoscopy, the risks of misplaced intubation in the esophagus were fewer than those in the direct method. Lewis (7) also stated no difference in hypoxia using two types of laryngoscopy methods although the video laryngoscopy reduces the chance of misplaced intubation. The finding of the present study regarding the lack of difference between two methods in the rate of misplaced intubation in the esophagus is inconsistent with that of other studies. However, in a clinical study carried out by Silverberg et al. (8) in 2015 on the assessment of video and direct laryngoscopy, no difference was observed between the two methods in terms of the rate of misplaced intubation in the esophagus. 

Intubation with Glide Scope allows the user to evaluate the laryngoscope on the monitor during training, which gives appropriate feedback. Lack of simultaneous observation of the mouth by two people (students and instructor) in direct laryngoscopy leads to the repetition of this process, waste of time in training, and lack of appropriate feedback. This significant difference between the two methods can lead to discrepancies in results obtained from the two groups. Various studies have shown that users who have not previously had an intubation experience achieved a success rate between 35% and 95% after 10 attempts of intubation. In addition, it is stated that an average of 57 times of intubation experience is needed to attain a 90% success rate. It is recommended that future studies include a bigger sample size, use other appropriate anesthetic drugs, and compare them together. Moreover, it is suggested to investigate other factors affecting the hemodynamic system of individuals. 

## Conclusion

In conclusion, video laryngoscopy performed by novice emergency medicine residents has a greater effect on hemodynamic variables of patients, compared to direct laryngoscopy; nevertheless, there is no difference between the two methods regarding the success rate of intubation and its complications. It is worth mentioning that there is no evidence to claim that the use of video laryngoscopy has reduced the incidence of hypoxia, respiratory complications, and frequency of attempts to intubate, or affected the time needed for intubation.
